# HUD Housing Assistance and Levels of Physical Activity Among Low-Income Adults

**DOI:** 10.5888/pcd15.170517

**Published:** 2018-07-19

**Authors:** Michelle S. Wong, Eric T. Roberts, Carolyn M. Arnold, Craig E. Pollack

**Affiliations:** 1 Department of Health Policy & Management, Johns Hopkins School of Public Health, Baltimore, Maryland; 2 Department of Health Policy & Management, University of Pittsburgh Graduate School of Public Health, Pittsburgh, Pennsylvania; 3 Johns Hopkins School of Medicine, Baltimore, Maryland; 4 Division of General Internal Medicine, Johns Hopkins School of Medicine, Baltimore, Maryland

## Abstract

**Introduction:**

Receipt of housing assistance from the US Department of Housing and Urban Development (HUD) is associated with improved health among adults and lower rates of unmet medical need among adults and young children. However, it is unclear whether HUD housing assistance is associated with healthier behaviors. The objective of our study was to assess whether participation in HUD housing assistance programs is associated with increased physical activity among low-income adults.

**Methods:**

In 2017, we pooled cross-sectional data from the 2004–2012 National Health Interview Survey (NHIS) linked to administrative records of HUD housing assistance participation. Our primary sample was low-income adults (aged ≥18; <200% of federal poverty level). Using multivariate logistic regression, we calculated the odds of being physically active (≥150 min/week of moderate-intensity activity or equivalent combination of moderate- and vigorous-intensity activity) among current HUD housing assistance residents compared with a control group of future residents (adults who would receive assistance within the next 2 years). In a secondary analyses, we examined neighborhood socioeconomic status as a modifier and conducted a subanalysis among nonsenior adults (aged <65).

**Results:**

Among all low-income adults, the adjusted odds of being physically active were similar for current and future residents (odds ratio =1.17; 95% confidence interval, 0.95–1.46). Among nonseniors, current residents were more likely to be physically active than future residents (odds ratio = 1.47; 95% confidence interval, 1.10–1.97). Associations did not differ by neighborhood socioeconomic status.

**Conclusion:**

Receiving HUD housing assistance is associated with being physically active among nonsenior low-income adults.

## Introduction

More than 5 million households receive federal assistance to help pay housing costs (1). The US Department of Housing and Urban Development (HUD) manages and oversees federal housing assistance programs, which can include the provision of housing vouchers (eg, Housing Choice Vouchers), which help people rent private homes, or the provision of public housing (2). The receipt of housing assistance is linked with better overall self-rated health, less psychological distress (3), and lower rates of uninsurance and unmet medical needs (4) among adults, as well as lower blood-lead levels in young children (5). The extent to which housing assistance may also lead to healthier behaviors, such as physical activity, is uncertain.

Understanding the link between housing assistance and physical activity is important because regular physical activity among adults confers numerous health benefits, including lower rates of cardiovascular disease, hypertension, type 2 diabetes, various cancers, and mortality (6–8). Less than half of low-income adults reach recommended physical activity goals (9).

Housing assistance may increase leisure-time physical activity by providing families with greater housing and resource stability, which in turn may enhance their ability to engage in health-promoting behaviors. Families that receive housing assistance pay no more than one-third of their household income on rent and utilities and move less often than those not receiving assistance (1,10). In contrast, low-income families without assistance frequently devote a substantial proportion of their incomes to housing expenses, and as a result, may need to move frequently, and are at a higher risk of becoming homeless (4). Costly and unstable housing environments could consume resources and time that could otherwise be used to support health promotion.

Aspects of the neighborhood environment, such as safety and aesthetics, may further influence the relationship between housing assistance and physical activity. Lower socioeconomic status (SES) neighborhoods typically have fewer physical-activity–promoting facilities (eg, parks) (11,12) and are more likely to be perceived as unsafe and unattractive (13), factors that may inhibit physical activity (11,13). Assisted households are often concentrated in lower-SES neighborhoods (14,15), which may limit the benefit of housing assistance on physical activity. Conversely, housing assistance programs that allow eligible people to move to higher-income neighborhoods may conceivably promote greater physical activity through an environment that has more physical-activity–promoting resources (16).

The primary objective of our study was to assess whether receiving HUD housing assistance is associated with physical activity levels in a national sample of US adults. We hypothesized that rates of physical activity would be higher among adults currently receiving housing assistance than among future housing assistance recipients. We had 2 secondary aims. First, we assessed whether neighborhood SES modified the association between housing assistance and adult physical activity levels. We hypothesized that the association between housing assistance and physical activity would be stronger among adults living in higher-SES neighborhoods. Second, because physical activity decreases with age (17,18), we examined the relationship between housing assistance and physical activity among a subset of nonsenior adults (aged <65). We hypothesized that rates of physical activity would be higher among nonsenior adults currently receiving assistance than among future housing assistance recipients.

## Methods

Data came from the National Health Interview Survey (NHIS) from 2004 through 2012; these data were linked to administrative data from HUD from 2004 through 2012 (19). The linked HUD administrative data provide information about whether and when NHIS respondents received HUD housing assistance. Linkage was limited to NHIS respondents who gave person-level identifiers and consented to future administrative linkage (52.5% of adult NHIS respondents). NHIS and HUD administrative data linkage was based on exact matches of a 9-digit Social Security number, sex, and month and year of birth. Neighborhood SES data came from the 2007–2011 American Community Survey 5-year estimates files (20); these data were linked to the NHIS data set by census-tract identifiers.

The sample for our primary analysis consisted of US adults (aged ≥18) who responded to the NHIS Sample Adult questionnaire, received HUD housing assistance at the time of the NHIS interview or would receive assistance within the next 24 months (average time on the wait-list), and lived in households with an income-to-poverty ratio less than 200% (n = 6,256).

### Measures

**Dependent variable.** Adults were categorized as being physically active if they met the US Department of Health and Human Services’ recommendation of 150 minutes or more of moderate-intensity aerobic physical activity per week or an equivalent combination of moderate- and vigorous-intensity aerobic activity (21). Respondents self-reported the frequency and duration of their usual moderate- and vigorous-intensity leisure-time physical activity; we summed minutes of moderate-intensity aerobic activity per week plus twice the minutes of vigorous-intensity aerobic activity per week to calculate total minutes of moderate-intensity–equivalent aerobic physical activity (22,23).

**Independent variables.** The main independent variable was a binary indicator of whether respondents currently received HUD housing assistance. We categorized respondents as receiving housing assistance if HUD administrative records indicated that they received housing assistance at the time of the NHIS interview (hereinafter termed “current assistance”).

A challenge of estimating the relationship between HUD housing assistance and health behaviors is unobserved confounding (ie, factors correlated with the receipt of housing assistance and physical activity [3]). To control for unobserved differences between adults with housing assistance and adults without housing assistance, we used a “pseudo–wait-list” approach. This approach compared adults who currently received housing assistance with adults who would receive housing assistance within 24 months after the NHIS survey (24 months is the average wait time for receiving housing assistance [24]). Wait-listed respondents likely resemble the current assistance group on numerous observed and unobserved characteristics. This pseudo–wait-list approach was used in previous studies to examine the association of housing assistance with health status and health care utilization (3,4). As a check on the suitability of this control group, we compared current recipients of assistance and future recipients of assistance on observed health and sociodemographic characteristics reported in NHIS.

**Effect modifiers.** To examine whether neighborhood SES modified the relationship between housing assistance and physical activity, we created a composite measure of neighborhood SES based on factors described in existing literature (25,26) and 6 variables describing neighborhood SES characteristics from the US Census that we identified through a factor analysis: 1) percentage of residents with a high school diploma or less, 2) percentage of residents with a 4-year college degree or less, 3) unemployment rate, 4) poverty rate, 5) percentage of working residents in a nonmanagement (primary) occupation, and 6) housing vacancy rate. We used factor loadings to create a census-tract–level neighborhood SES index. We then categorized census tracts into quartiles from highest SES (quartile 1) to lowest SES (quartile 4).

**Covariates.** We controlled for the following respondent characteristics: age; sex; race/ethnicity; family size; number of functional limitations (number of the following activities that respondents reported “very difficult” or “unable to” do: walk a quarter-mile, walk up 10 steps, stand for 2 hours, sit for 2 hours, stoop/bend/kneel, reach overhead, grasp small objects, lift/carry up to 10 pounds, or push/pull large objects, as consistent with previous studies [27,28]); education level of person in household with highest level of education; family income-to-poverty ratio; employment status; receipt of Supplemental Nutrition Assistance Program, Special Supplemental Nutrition Program for Women, Infants, and Children, and/or Temporary Assistance for Needy Families benefits; marital status; self-rated health status; and neighborhood SES. We also included a state and survey year interaction term to control for temporal trends in state-level characteristics that might affect physical activity levels or receipt of housing assistance (eg, regional differences in investment in physical-activity–promoting resources or in state-level housing assistance policies).

### Statistical analysis

In 2017, we calculated summary statistics of means and proportions for all variables stratified by housing assistance status. For our primary aim, we used multivariate logistic regression to assess the relationship between receipt of HUD housing assistance (comparing current and future recipients) and physical activity among all low-income adults (aged ≥18), controlling for respondent-level variables listed above and neighborhood SES.

To test for effect modification by neighborhood SES, we ran a separate multivariate logistic regression model that included an interaction between housing assistance status and neighborhood SES while controlling for all respondent-level variables in the main analysis. We used an *F* test to assess whether interactions between housing assistance receipt and neighborhood SES categories were jointly significant.

To examine the association between housing assistance and physical activity among nonseniors, we repeated our analyses in a subset of nonsenior adults (aged <65) while controlling for all individual and neighborhood SES characteristics as in the main analysis. We also examined effect modification by neighborhood SES among nonseniors through a separate model in this subgroup that included the housing assistance–neighborhood SES interaction while controlling for respondent-level characteristics.

All estimates were weighted to account for the NHIS sampling frame and linkage eligibility of respondents. Design-based variance estimates were used to account for the NHIS’s complex survey design. Because the rate of missingness was low among our analytic sample (<1% for any variable), we excluded respondents with missingness for any covariates. Statistical analyses were performed in Stata/IC 14.1 (StataCorp LLC).

### Sensitivity analysis

We performed several sensitivity analyses, including 1) re-running our analyses with physical activity as a continuous variable and 2) including individuals living in households with an income-to-poverty ratio greater than 200%. Additionally, consistent with a previous study of this population (5), we used a propensity-score approach to compare physical activity among adults who received housing assistance when surveyed to a comparable group of adults not receiving housing assistance at that time. The propensity-score approach allowed for a larger control group than did the pseudo–wait-list, because it was not limited to people who eventually received housing. However, propensity scores can balance individuals only on observed characteristics. We used the inverse probability of treatment weights to weight the sample of low-income adults not receiving housing assistance to resemble the sample of assisted adults on observed characteristics. We ran separate propensity-score sensitivity analyses among all adults and in the nonsenior subpopulation (Appendix).

## Results

The sample of 6,256 surveyed low-income adults represented 6,472,700 adults receiving current and future HUD housing assistance in the United States. Compared with adults receiving future assistance, adults receiving current assistance were significantly older (46.3 y vs 42.4 y), more likely to be female (73.8% vs 69.6%), more likely to live in a household with an income-to-poverty ratio less than 100% (69.4% vs 60.9%), less likely to be currently married (14.6% vs 20.3%), and less likely to be currently employed (27.3% vs 33.4%); they had more functional limitations (1.6 vs 1.3 limitations) and a smaller family size (2.4 vs 2.8 family members) (Table 1).

**Table 1:** Characteristics of a Sample of Low-Income Adults (Aged ≥18), by HUD Housing Assistance Status, in a Study of HUD Housing Assistance and Levels of Physical Activity Among Low-Income Adults, 2004–2012^a^

Characteristic	Receiving Current Assistance (n = 5,233)	Would Receive Future Assistance^b^ (n = 1,023)	*P* Value^c^
**Meets criteria for being physically active^d^**	24.8	24.9	.98
**Minutes of moderate-intensity–equivalent aerobic physical activity, mean (SD)**	181.2 (822.5)	177.1 (637.1)	.88
**Age, mean (SD), y**	46.3 (22.8)	42.4 (22.8)	<.001
**Female sex**	73.8	69.6	.03
**Race/ethnicity**			
Non-Hispanic white	39.0	40.5	.57
Non-Hispanic black	35.6	36.8	
Hispanic	18.6	17.1	
Non-Hispanic other	6.8	5.6	
**Education level of person in household with highest level of education**			
<High school diploma	30.0	27.7	.22
High school diploma	64.0	67.6	
≥4-year college degree	5.9	4.8	
**Family income-to-poverty ratio**			
<0.50	23.7	22.4	<.001
0.50–0.99	45.8	38.5	
1.00–1.49	23.2	25.2	
1.50–1.99	7.4	13.9	
**Currently employed**	27.3	33.4	.004
**Receives any federal assistance^e^**	65.2	68.6	.11
**Marital status**			
Never married	45.0	46.8	<.001
Currently married	14.6	20.3	
Previously married	40.4	32.9	
**No. of family members in household, mean (SD)**	2.4 (2.1)	2.8 (2.2)	<.001
**Self-rate health**			
Excellent/very good	30.7	32.7	.36
Good/fair/poor	69.3	67.3	
**No. of functional limitations^f^, mean (SD)**	1.6 (3.3)	1.3 (2.6)	.004
**Neighborhood socioeconomic status**			
Quartile 1 (highest)	17.4	17.3	.98
Quartile 2	24.5	23.8	
Quartile 3	27.8	28.6	
Quartile 4	30.3	30.4	

Abbreviations: HUD, US Department of Housing and Urban Development; NHIS, National Health Interview Survey; SD, standard deviation.

a Data sources: NHIS 2004–2012; these data were linked to administrative data from HUD 2004–2012 (19). Neighborhood socioeconomic data came from the 2007–2011 American Community Survey 5-year estimates files (20); these data were linked to the NHIS data set by census-tract identifiers. Proportions and means were calculated by using survey weights provided by NHIS. Unless otherwise indicated, values presented are percentages.

b Adults who would receive housing assistance within 24 months after the NHIS survey.

c Determined by *t* test for means and χ^2^ test for proportions.

d 150 minutes or more of moderate-intensity aerobic physical activity per week or an equivalent combination of moderate- and vigorous-intensity aerobic activity (21).

e Supplemental Nutrition Assistance Program; Special Supplemental Nutrition Program for Women, Infants, and Children; and/or Temporary Assistance for Needy Families.

f Activities that respondents reported “very difficult” or “unable to” do: walk a quarter-mile, walk up 10 steps, stand for 2 hours, sit for 2 hours, stoop/bend/kneel, reach overhead, grasp small objects, lift/carry up to 10 pounds, or push/pull large objects.

Overall, 25% of adults in both current and future assistance groups met the criteria for being physically active (Table 1). After adjusting for respondent characteristics and neighborhood SES, adults receiving current housing assistance had a higher adjusted odds of being physically active compared with adults receiving future housing assistance, although this relationship was not significant (odds ratio [OR] = 1.17; 95% confidence interval [CI], 0.95–1.46) (Figure).

**Figure:**
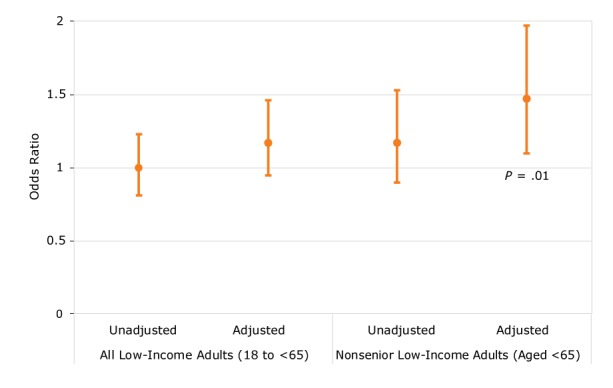
Association between current HUD housing assistance status versus future HUD housing assistance status and being physically active among all low-income adults (aged ≥18) and nonseniors (aged <65) in the United States, 2004–2012 National Health Interview Survey. **Table:** 

Population/Model	Odds Ratio (95% Confidence Interval)
All low-income adults	
Unadjusted	1.00 (0.81–1.23)
Adjusted	1.17 (0.95–1.46)
Nonseniors	
Unadjusted	1.17 (0.90–1.53)
Adjusted	1.47 (1.10–1.97)

We did not find evidence that neighborhood SES modifies the association between housing assistance and being physically active among low-income adults (interaction *P* = .44).

Our subsample of 3,933 surveyed nonseniors represented a weighted sample of 4,086,754 adults. Among all nonseniors, 26% were physically active, including 27% of those currently receiving housing assistance and 24% of those receiving future housing assistance (*P* = .23) (Table 2). In adjusted analyses, current housing assistance was significantly associated with increased odds of physical activity compared with future housing assistance (OR = 1.47; 95% CI, 1.10–1.97; *P* = .01) (Figure). We did not find evidence of effect modification by neighborhood SES among low-income nonsenior adults (interaction *P* = .34).

**Table 2:** Characteristics of a Sample of Nonsenior (Aged 18 to <65) Low-Income Adults, by HUD Housing Assistance Status, in a Study of HUD Housing Assistance and Levels of Physical Activity Among Low-Income Adults, 2004-2012^a^

Characteristic	Receiving Current Assistance (n = 3,268)	Would Receive Future Assistance^b^ (n = 665)	*P* Value^c^
**Meets criteria for being physically active^d^**	27.1	24.0	.23
**Minutes of moderate-intensity–equivalent aerobic physical activity, mean (SD)**	212.2 (1,212.6)	161.1 (558.1)	.09
**Age, mean (SD), y**	38.3 (13.9)	35.5 (12.6)	<.001
**Female sex**	75.0	72.2	.26
**Race/ethnicity**			
Non-Hispanic white	31.7	32.8	.94
Non-Hispanic black	42.7	42.5	
Hispanic	19.5	19.4	
Non-Hispanic other	6.1	5.3	
**Education level of person in household with highest level of education**			
<High school diploma	24.8	25.6	.08
High school diploma	70.0	71.7	
≥4-year college degree	5.1	2.6	
**Family income-to-poverty ratio**			
<0.50	29.7	24.9	<.001
0.50–0.99	44.3	38.5	
1.00–1.49	19.1	22.4	
1.50–1.99	6.9	14.2	
**Currently employed**	34.1	39.2	.06
**Receives any federal assistance^e^**	76.0	72.0	.11
**Marital status**			
Never married	55.33	54.7	.03
Currently married	14.8	19.5	
Previously married	29.9	25.8	
**No. of family members in household, mean (SD)**	2.8 (1.6)	3.2 (1.7)	<.001
**Self-rate health**			
Excellent/very good	34.1	36.5	.40
Good/fair/poor	65.9	63.5	
**Number of functional limitations^f^, mean (SD)**	1.2 (3.2)	1.1 (2.4)	.09
**Neighborhood socioeconomic status**			
Quartile 1 (highest)	14.0	13.3	.86
Quartile 2	23.1	21.6	
Quartile 3	28.4	30.7	
Quartile 4	34.5	34.5	

Abbreviations: HUD, US Department of Housing and Urban Development; NHIS, National Health Interview Survey; SD, standard deviation.

a Data sources: NHIS 2004–2012; these data were linked to administrative data from HUD 2004–2012 (19). Neighborhood socioeconomic data came from the 2007–2011 American Community Survey 5-year estimates files (20); these data were linked to the NHIS data set by census-tract identifiers. Proportions and means were calculated by using survey weights provided by NHIS. Unless otherwise indicated, values presented are percentages.

b Adults who would receive housing assistance within 24 months after the NHIS survey.

c Determined by *t* test for means and χ^2^ test for proportions.

d 150 minutes or more of moderate-intensity aerobic physical activity per week or an equivalent combination of moderate- and vigorous-intensity aerobic activity (21).

e Supplemental Nutrition Assistance Program; Special Supplemental Nutrition Program for Women, Infants, and Children; and/or Temporary Assistance for Needy Families.

f Activities that respondents reported “very difficult” or “unable to” do: walk a quarter-mile, walk up 10 steps, stand for 2 hours, sit for 2 hours, stoop/bend/kneel, reach overhead, grasp small objects, lift/carry up to 10 pounds, or push/pull large objects.

### Sensitivity analysis

When we coded physical activity as a continuous variable, housing assistance was not associated with physical activity in the overall sample. Among nonsenior adults, the association between physical activity and housing assistance was not significant (*P* = .06). Including respondents who lived in households with an income-to-poverty ratio above 200% did not alter our main findings.

In propensity-scored weighted analyses, the odds of being physically active did not differ between adults receiving current housing assistance and adults not receiving current assistance (OR = 0.94; 95% CI, 0.84–1.04) and the nonsenior subgroup (OR = 1.01; 95% CI, 0.92–1.10). Neighborhood SES did not modify these associations among all low-income adults (interaction *P* = .88) and nonseniors (interaction *P* = .52).

## Discussion

We found that among low-income nonseniors younger than 65, current HUD housing assistance recipients were more likely to meet recommended physical activity goals than were nonseniors receiving future assistance, whereas among all low-income adults in our sample, we found insufficient evidence of a difference in physical activity by HUD housing assistance status. To our knowledge, our study is the first to assess whether receiving HUD housing assistance is associated with health behaviors — specifically, physical activity — in a nationally representative sample of adults in the United States.

The difference in our findings between all low-income adults and the nonsenior subgroup suggests that the benefits of receiving HUD housing assistance on increased physical activity is primarily among nonsenior adults. Reasons for these difference warrant further investigation. Although receipt of HUD housing assistance might increase the financial resources of both younger and older adults, these 2 groups of adults may differently direct these resources. For example, because seniors generally have a higher burden of chronic disease, they may apply any increases in financial resources toward medical needs rather than physical activity. Although differences in our findings between younger adults and our overall sample may stem from differences in functional status, our analyses controlled for self-rated health and functional limitations. Another potential factor may be differences in housing assistance programs and facilities available to younger and older adults (eg, senior housing) that may affect levels of physical activity. Given the numerous health benefits of physical activity, particularly in preventing chronic conditions that increase in incidence and severity with age, increasing physical activity in nonsenior adults is an important public health priority.

Our finding of no difference in the relationship between HUD housing assistance and physical activity by neighborhood SES is surprising given evidence that neighborhood SES can influence physical activity through the availability of physical-activity–promoting facilities and perceived neighborhood attractiveness and safety (11–13). It is possible that, although higher-SES neighborhoods might have more physical activity facilities, they might not offer affordable options (eg, free or low-cost recreational centers that are more common in lower-SES neighborhoods). Additionally, receiving housing assistance in a higher-SES neighborhood might require adults to move, which disrupts important social networks that can encourage physical activity (eg, walking groups) (29). However, the lack of modification by neighborhood SES in our analysis is consistent with a study that examined the relationship between HUD housing assistance and adult health (3).

Our results should be interpreted with caution because results from our main analysis (the pseudo–wait-list approach) differed from the propensity-score sensitivity analysis. However, we believe that the lack of association in propensity-score sensitivity analyses might be due to potential unobserved differences between assisted and unassisted individuals that we could not adequately account for with the available data. When we assessed balance between current recipients and the propensity-weighted comparison group, we found some differences between groups. Adding other covariates available in NHIS could not substantially improve balance between groups. We acknowledge, though, that the pseudo–wait-list approach may also be vulnerable to confounding and selection bias (3,4). Despite this, the comparison groups in the pseudo–wait-list approach — used in previous analyses of HUD housing assistance with this same data set (3,4) — were generally similar on observed characteristics included in our analysis, which increases our confidence in the pseudo–wait-list results. Another reason for caution is the lack of significant association between housing assistance and physical activity coded as a continuous variable among nonseniors.

Our study has several limitations. First, we were unable to explicitly compare current assistance residents to those known to be on the HUD wait-list, because wait-list data are not available for all HUD program types. Individuals not on the actual wait-list but included in our pseudo–wait-list may have experienced a change (eg, disability, job loss) that might have simultaneously induced them to apply for housing assistance and affected their physical activity. However, a study that examined adult health status outcomes found that results from the pseudo–wait-list and the available wait-list data were nearly identical (3). We also used a 2-year time period for determining the wait-list, recognizing that the wait-list times vary among jurisdictions. Second, we assumed that wait-list individuals were comparable to current assistance recipients on all observed and unobserved characteristics, as in previous housing and health studies (30). Third, our measure of vigorous physical activity and other covariates was self-reported, and these data may be prone to recall and social desirability bias. The rates of physical activity we found were similar to those found in other national surveys conducted among adults (31), and we do not have evidence to suggest that systematic reporting errors would exist between adults based on their housing assistance status. Fourth, our neighborhood measure was designed to assess overall socioeconomic status, and we were unable to access data on specific features of neighborhoods (eg, parks) that are linked with physical activity. Fifth, we relied on the HUD data linkage using exact matches on numerous personal identifiers. Incomplete data linkage may potentially bias findings. Fifth, although receipt of Supplemental Security Income or Social Security Disability Insurance may influence receipt of HUD assistance, we were unable to control for either of these, which may have biased our results toward the null. Sixth, we dichotomized physical activity based on recommended guidelines, but this approach may have resulted in a loss of information. Lastly, we were underpowered to determine whether differences existed by HUD assistance program type.

As is well-documented, rates of physical activity among US adults are suboptimal. This study suggests that receiving HUD housing assistance is associated with increased levels of physical activity among low-income nonsenior adults. Along with previous research that examined the relationship between housing assistance and health conditions and health care utilization, our findings indicate that housing may also be a platform for healthy behaviors, which may, in turn, have long-term effects across a range of conditions. Programs and initiatives that seek to improve physical activity overall may consider the important role of housing assistance in health promotion.

## Appendix Description of Propensity-Score Sensitivity Analysis

As a sensitivity analysis, we used propensity-score estimation to compare the effect of housing assistance on physical activity comparing adults who received housing assistance at the time of the survey to a comparable group of adults not receiving housing assistance at that time. Use of propensity scores allowed us to better control for confounding between housing assistance–eligible adults who did and did not receive housing assistance and physical activity level compared to multivariate regression comparing those who currently receive assistance with those who currently do not receive assistance (regardless of future assistance status). The estimand of interest for this analysis is the population average treatment effect on the treated, which provides us an estimate of the effect of housing assistance on physical activity level only on those who receive housing assistance.

Using logistic regression, we estimated the propensity score for adults receiving housing assistance compared to those not receiving housing assistance. In addition to all the confounding variables described above, we also included the NHIS-provided survey weights as predictors in estimating the propensity-score weights. We trimmed weights at the 95th percentile of the distribution to avoid extreme weights. We examined propensity-score distributions between the housing assistance and non-housing assistance groups with histograms to ensure sufficient overlap between both groups.

We matched the housing assistance and non-housing assistance groups using the weighting by the odds method. The housing assistance group received a weight of 1 and the non-housing assistance group received a weight of the odds of the propensity score (ie, PS/[1 − PS]). We then created a composite of the propensity weight with the survey weight by multiplying the two together. We calculated and compared standardized biases before and after propensity-score weighting to assess covariate balance. For the outcome model, we ran a weighted logistic regression that controlled for all the potential confounding variables as described in the Measures section, and weighted the analysis with the combined propensity and survey weights.
